# RECRUITMENT OF THE CENTRAL NERVOUS SYSTEM IN DIFFERENT HAND TASKS IN PATIENTS WITH HAND DYSFUNCTION AFTER STROKE BASED ON FUNCTIONAL NEAR-INFRARED SPECTROSCOPY: AN EXPLORATORY STUDY

**DOI:** 10.2340/jrm.v58.44712

**Published:** 2026-03-09

**Authors:** Ning ZHANG, Haolin TIAN, Yuanbin YANG, Qinxuan SHEN, Ziyi LI, Long HE, Jing ZHOU, Xuechao LI, Jingfeng TIAN, Mengying WAN, Wei YAO, Longyue YI

**Affiliations:** 1 Department of Rehabilitation Medicine, Wangjing Hospital of China Academy of Chinese Medical Sciences, Beijing, China; 2 Department of Rehabilitation Medicine, Beijing Tongren Hospital, Capital Medical University, Beijing, China; 3 School of Sports Medicine and Rehabilitation, Beijing Sport University, Beijing, China

**Keywords:** stroke, hand dysfunction, fNIRS, rehabilitation, neuroplasticity

## Abstract

**Objective:**

This study aimed to examine the central nervous system activation in stroke patients with hand dysfunction during various hand tasks, reflecting central nervous system recruitment.

**Design:**

A single-centre cross-sectional observational study.

**Patients:**

This research selected stroke patients with hand dysfunction hospitalized in the authors’ hospital from October 2022 to November 2023. Participants were aged 25–75 years, with a post-stroke duration ranging from 2 to 24 weeks.

**Methods:**

A 35-channel functional near-infrared spectroscopy system was used to record cortical activity during the resting state, affected-hand grasping tasks, and hand-crank cycling tasks. The study compared the average brain activation extent and functional connectivity between grasping and handbike tasks, focusing on the primary sensorimotor cortex, dorsolateral prefrontal cortex, primary motor cortex, and primary somatosensory cortex as regions of interest.

**Results:**

Comparative analysis of brain region activation revealed significant increases in activation across all regions of interest compared with the resting state (*p <* 0.001). When comparing grasping with handbike tasks, significant increases in activation were observed in all regions of interest except the right primary somatosensory cortex (*p* < 0.05). Additionally, the right dorsolateral prefrontal cortex exhibited stronger functional connectivity with bilateral primary motor cortex, primary sensorimotor cortex, and left primary somatosensory cortex during the grasping task compared with the handbike task (*p* < 0.05).

**Conclusion:**

This study shows that grasping tasks recruit cognitive, sensory, and motor cortex activities in stroke patients with hand dysfunction relatively higher than handbike tasks.

## LAY ABSTRACT

After a stroke, many people have difficulty using their hand. We used a safe, portable brain scanner called fNIRS to compare 2 exercises: repeatedly opening and closing the hand, and using an arm-cycling machine. We found that both activities increased brain activity, but the hand movement stimulated more areas involved in motion, feeling, and thinking, and helped different brain regions communicate better. This suggests that simple hand opening and closing practice may be especially helpful for brain recovery after a stroke. Our results can assist therapists in choosing more effective rehabilitation methods.

Stroke is the third leading cause of death globally (1), and induces a spectrum of dysfunctions, encompassing motor impairment, sensory deficits, and cognitive decline, among others. Notably, over 70% of stroke patients experience upper limb motor dysfunction (2–4). Daily activities necessitate intricate hand motor tasks, with dexterity proficiency, defined as adeptness in grasping and manipulating objects through precise hand and finger coordination, closely linked to independence in daily living (5). After a stroke, neuronal circuit disruption causes upper limb muscular denervation, abnormal spasticity and movement patterns, as well as decreased muscle strength, which particularly affects distal hand and finger recovery (6). Presently, hand dysfunction post-stroke is predominantly addressed through conventional rehabilitation techniques such as physical and occupational therapy, targeting daily life skills. These therapies entail standard hand movements along with specific tasks. However, current clinical interventions lack precise elucidation of motor control mechanisms, notably at the central system level, and lack dynamic, objective, and quantitative rehabilitation evaluation metrics (7, 8). Functional neuroimaging studies in stroke patients with hand dysfunction primarily focus on cortical activation during simple tasks like grasping or assisting grasping, necessitating further comparative investigations into the cortical activation effects of commonly employed upper limb rehabilitation protocols.

Previous studies have shown that motor control of the hand is primarily associated with the primary sensorimotor cortex (SM1), dorsolateral prefrontal cortex (DLPFC), primary motor cortex (PMC), and primary somatosensory cortex (S1) (9–12). SM1 and S1 are linked to sensorimotor integration, while DLPFC is implicated in cognitive functions, including working memory, cognitive processing, motor control, attention allocation, and inhibitory control. PMC directly influences motor behaviour (13). When stroke patients with hand dysfunction engage in various upper limb tasks, they recruit different cognitive, motor, sensory, and other functions. Consequently, the areas activated by the central nervous system, the extent of activation, and the strength of functional connectivity differ, resulting in distinct brain regulation effects.

Functional near-infrared spectroscopy (fNIRS) represents a novel non-invasive neuroimaging technology that exhibits a significant correlation with the blood oxygen level-dependent response obtained via fMRI. Together, they have emerged as effective tools for exploring the internal functional organization of the human brain (14). Compared with fMRI, fNIRS offers advantages such as safety, portability, minimal restriction, mobility, repeatability, low cost, resilience to motion artefacts, higher temporal resolution, and enhanced patient acceptance. Particularly suitable for bedside and real-time measurement scenarios, it holds potential for widespread application in neuroscience research, especially among particular populations like the elderly, children, and patients with various diseases (15–18). Stroke patients with hand dysfunction often exhibit motion artefacts during hand tasks due to spasticity and diminished muscle strength. Moreover, some patients with mobility issues require bedside monitoring and long-term real-time measurement of hand task performance. Thus, fNIRS is well suited as a research tool for investigating central nervous system recruitment in this study.

The purpose of this study is to explore the central nervous system response in stroke patients with hand dysfunction during various hand tasks, reflecting central nervous system recruitment, and to offer insights for the training and evaluation of these patients.

## METHODS

### Study design

This study adopts a single-centre cross-sectional observational design, conducted at our hospital from October 2022 to November 2023.

### Participants

The study included patients with hand dysfunction after stroke hospitalized at our hospital during the specified period. Inclusion criteria were as follows: (*i*) Meeting the diagnostic criteria for cerebral haemorrhage or cerebral infarction according to the 2019 Major Cerebrovascular Diseases diagnostic criteria issued by the Chinese Society of Neurology; (*ii*) aged between 25 and 75 year; (*iii*) within 2 to 24 weeks post-stroke with stable condition and tolerance for rehabilitation treatment; (*iv*) experiencing upper limb dysfunction due to stroke; (*v*) voluntarily participating in the clinical trial and providing informed consent. Exclusion criteria were: (*i*) hemiplegia from non-cerebrovascular conditions such as brain trauma, tumour, or craniocerebral surgery; (*iii*) co-occurrence of cerebral infarction and cerebral haemorrhage; (*iii*) severe heart, lung, liver, kidney, or other organ failure; (*iv*) severe aphasia, auditory comprehension, or cognitive dysfunction hindering communication; (*v*) history of previous stroke and/or residual limb dysfunction; (*vi*) upper limb paralysis from factors other than stroke, such as Guillain-Barré syndrome, multiple sclerosis, or myasthenia gravis; (*vii*) poor compliance with medical advice; (*viii*) previous receipt of systematic rehabilitation therapy.

**Fig. 1:**
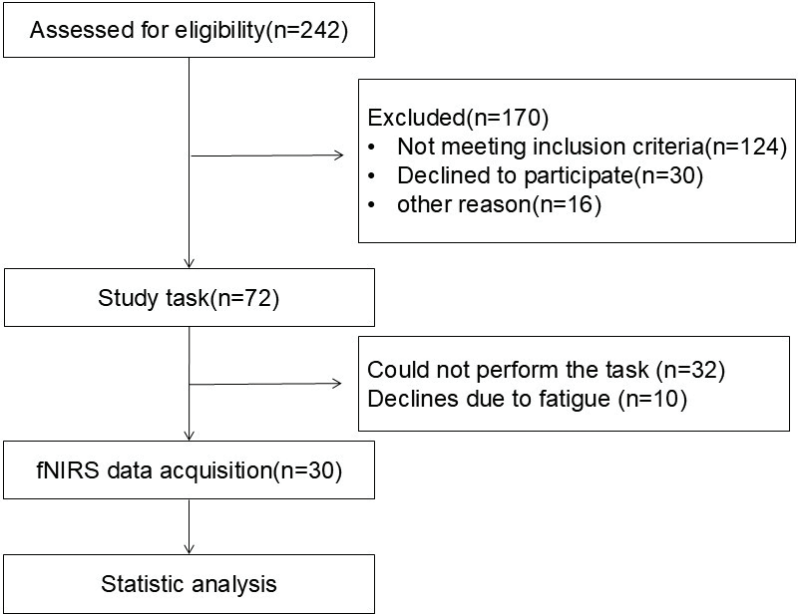
Flowchart of the study participants.

Ethics approval and consent were obtained from the Ethics Committee of our Hospital. The participant enrolment process for the study is shown in Fig. 1.

### Imaging-fNIRS

**Fig. 2:**
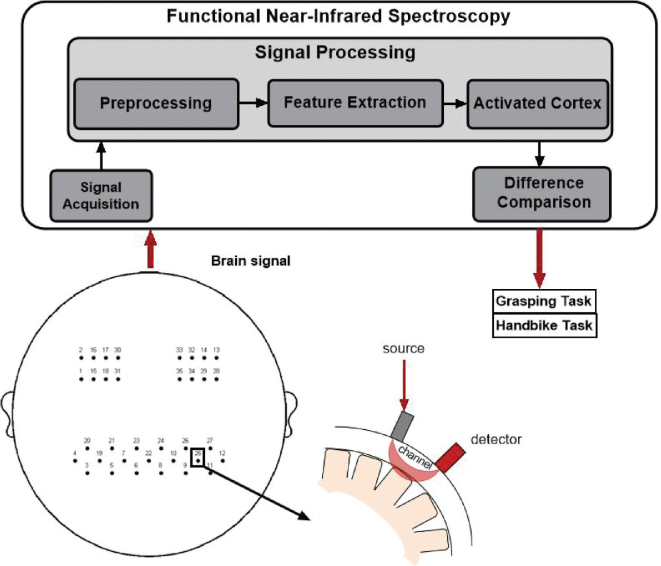
General schematic of the different components of fNIRS measurement.

The fNIRS imaging device NirSmart (Danyang Huichuang Medical Equipment Co, Shaanxi, China) was utilized to capture the haemodynamic signals of subjects during the resting state, grasping task, and handbike task. Following the international 10–20 EEG placement system, 35 fNIRS channels were positioned in key brain regions such as the motor cortex, frontal cortex, and temporal cortex (Fig. 2). Near-infrared light (730,850 nm) signals of 2 distinct wavelengths were recorded in continuous wave format, with a sampling frequency of 11 Hz. The optical probe consisted of 14 transmitters and 14 receivers, with a 3 cm interval between the illuminant and detector. Real-time detection of changes in Deoxy-Hb and Oxy-Hb concentrations in the subjects’ brains was enabled. During the affected hand grasping task, subjects were instructed to relax, place the affected hand on their thigh or a table, and repetitively flex and extend their fingers without inducing body shaking. The handbike task required subjects to maintain a stable grip, holding the hand-pedal with both hands, and performing hand-pedalling at a comfortable speed without interference. Prior to measurements, subjects underwent several practice sessions for training. Hand-cranked bicycle training requires the subject to be seated and facing the machine comfortably, holding the handles on both sides firmly with both hands to maintain a stable grip. After hearing the standardized “start” command, the subject is required to synchronously drive both upper limbs at a comfortable speed, performing smooth and alternating push–pull movements along a circular trajectory. This training focuses on strengthening the rhythmic flexion and extension of the shoulder and elbow and wrist coordination, so as to complete the upper limb training characterized by gross movement and bilateral coordination. Specifically, each session involving 3 task paradigms required approximately 20 min, including fNIRS setup. Prior to formal data acquisition, participants were thoroughly instructed on the experimental procedures, and multiple practice trials were conducted to ensure smooth task performance. During the hand-crank cycling task, participants were instructed to maintain a pace of 1 revolution every 3–5 s. An examiner manually monitored the movement speed and provided reminders when necessary.

### Study task

We deliberately selected 2 rehabilitation-related tasks with distinct contrasts: a unilateral fine motor grasp task and a bilateral rhythmic gross motor hand-cranking task. Through this comparison, we can explore how the central nervous system differentially allocates resources according to task differences in limb side, complexity, and motor control demands, which is crucial for understanding task-specific neural plasticity and optimizing personalized rehabilitation programmes.

All tasks were conducted in a quiet room under 1:1 supervision. Subjects were seated comfortably in front of a computer screen and familiarized with the tasks before the formal test to grasp the experimental process and prevent unnecessary movements. During the formal test, subjects wore the portable fNIRS device head cap, positioning its leading edge above the brow bone. Successively, subjects completed the resting state measurement, affected hand grasping task, and handbike task. Task instructions were delivered through computer voice prompts. The experimental paradigm is depicted in Fig. S1. Task specifics were as follows.

Initially, for activation quantification, we computed ΔOxy-Hb during the resting state for each ROI. Subjects were instructed to relax, sit quietly, avoid movement, and refrain from regular thinking, while 5 min of resting state data (19) were collected to assess central nervous system activity.

*Task 1: Grasping with the affected hand.* This task comprised 6 blocks, with each block involving 40 s of grasping followed by 20 s of rest, totalling 1 min. Upon hearing the computer’s “exercise” command, subjects initiated repeated grasping actions with the affected hand while keeping the healthy hand still. The entire process lasted 360 s.

*Task 2: handbike task.* Similarly, this task consisted of 6 blocks, each featuring 40 s of handbike activity followed by a 20-s rest, totalling 1 min. Upon hearing the computer’s “exercise” command, subjects commenced handbike activity. The entire process lasted 360 s (see Fig. S2).

### fNIRS data pre-processing

Pre-processing was conducted using NirSpark analysis software (Danyang Huichuang, China). A wavelet-based approach was employed to remove irrelevant time intervals and artefacts induced by motion and environmental factors (20). Light intensity was converted to optical density and blood oxygen concentration using a modified Beer–Lambert law (21). Noise and interfering signals such as heart rate, respiratory rate, and Mayer waves were then eliminated using a bandpass filter (0.01–0.1 Hz). Based on coordinate information, some of the 35 channels were categorized into 4 regions of interest (ROI) of the left and right brain, namely SM1, DLPFC, PMC, and S1 (Table SI). The change in Oxy-Hb concentration during each task was averaged. The initial time of the haemodynamic response function (HRF) was set at –2 s (baseline state), and the end time was set at 40 s (single-block task state). Oxygenated HRF was averaged for each brain region across the 8 blocks. Finally, the generalized linear model was employed to identify significantly activated brain regions.

### Functional connectivity analysis

Functional connectivity analysis was conducted using NirSpark software, analysing the Pearson correlation coefficient (*r* value) of oxy-Hb and Deoxy-Hb concentration in each channel on the time series, followed by Fisher z-transformation. The Z-score post-transformation was defined as the functional connectivity strength between channels, and the functional connectivity map of the data was generated using the software.

### Statistical analysis

SPSS 26.0 statistical software (IBM Corp, Armonk, NY, USA) was utilized for statistical analysis. Data with normal distribution were expressed as mean ± standard deviation, while data with non-normal distribution were described using median and interquartile range. Paired-sample rank sum tests were employed for intergroup comparisons. The differences in channel activation during resting state, grasping, and handbike tasks were calculated and compared, respectively. Paired-sample *t*-tests were used to compare the mean Z-scores of functional connectivity strength across different tasks on ROI time series using NirSpark software. A significance level of *p <* 0.05 was considered statistically significant.

## RESULTS

### Baseline characteristics

This study enrolled a total of 30 patients with hand dysfunction following stroke, comprising 21 males and 9 females. The age ranged from 42 to 71 years, with disease durations spanning 2 to 20 weeks. Among the participants, 2 cases involved cerebral haemorrhage, while 28 cases presented with cerebral infarction. Left hemiplegia was observed in 14 cases, while right hemiplegia was present in 16 cases. Table I summarizes the baseline data of stroke patients, including age, gender, lesion type, lesion location, and disease duration. Table II presents the detailed lesion locations of the 30 enrolled patients.

**Table I:** Basic information on the participants

Demographics and descriptors	Subject
*n*	30
Age(years)	61.7 ± 7.7
Sex (m:f)	21:9
Stroke type (ICH:CI)	2:28
Stroke location (r:l)	12:18
Time since stroke (weeks)	8.1 ± 5.6
Fugl-Meyer-UP	35.64 ± 18.56

ICH: ischaemic; CI: cerebral infarction; ; Fugl-Meyer-UP: Fugl-Meyer Assessment-Upper Extremity.

**Table II:** Specific lesion locations in 30 subjects

Stroke location	*n*	Total
ICH		2
Left basal ganglia	1	
Right basal ganglia	1	
CI		28
Left pons	3	
Left brainstem	1	
Left basal ganglia	5	
Left basal ganglia and adiation crown	2	
Left frontal lobe, pons, and cerebellum	1	
Left frontal lobe, parietal lobe, temporal lobe, and insula	1	
Left frontal lobe, parietal lobe, and basal ganglia	1	
Left parietal lobe and periventricular region	1	
Left periventricular region	2	
Right pons	4	
Right brainstem	1	
Right basal ganglia and radiation crown	1	
Right basal ganglia	1	
Right radiation crown and subcortical area of the parietal lobe	1	
Right parietal lobe, temporal lobe, occipital lobe, and thalamus	1	
Right periventricular region and semiovoid centre	1	
Right periventricular region	1	

ICH: ischaemic; CI: cerebral infarction.

### Comparison of brain activation under resting state and grasping task

During the grasping task, significant increases in ΔHbO values compared with the resting state were observed in various brain regions, including left SM1 (Z = 18.892, *p* < 0.001), right SM1 (Z = 19.208, *p* < 0.001), left DLPFC (Z = 21.236, *p* < 0.001), right DLPFC (Z = 17.381, *p* < 0.001), left PMC (Z = 22.238, *p* < 0.001), right PMC (Z = 18.493, *p* < 0.001), left S1 (Z = 19.145, *p* < 0.001), and right S1 (Z = 19.684, *p* < 0.001) (refer to Table SII). All observed differences were statistically significant.

### Comparison of brain activation under resting state and handbike task

During the handbike task, notable increases in ΔHbO values compared with the resting state were observed across various brain regions, including left SM1 (Z = 17.331, *p* < 0.001), right SM1 (Z = 19.938, *p* < 0.001), left DLPFC (Z = 9.974, *p* < 0.001), right DLPFC (Z = 7.284, *p* < 0.001), left PMC (Z = 21.576, *p* < 0.001), right PMC (Z = 18.040, *p <* 0.001), left S1 (Z = 17.709, *p* < 0.001), and right S1 (Z = 19.617, *p* < 0.001) (refer to Table SIII). All observed differences were statistically significant.

### Comparison of brain activation under grasping task and handbike task

**Fig. 3:**
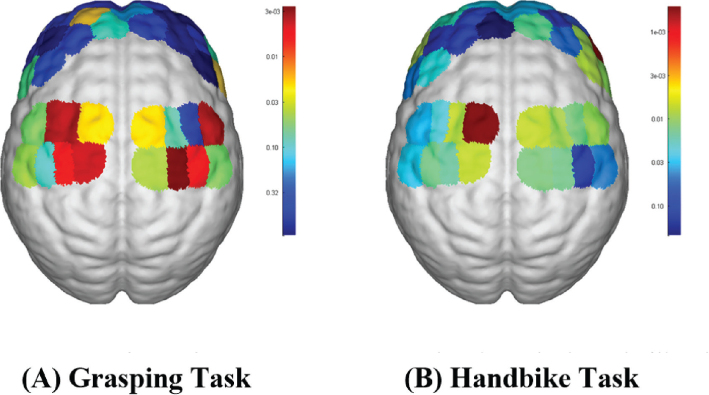
fNIRS activation maps for the 2 hand tasks: (A) grasping task (B) handbike task.

During the grasping task, significantly higher ΔHbO values compared with the handbike task were observed across various brain regions, including left SM1 (*p* = 0.048), right SM1 (*p* < 0.001), left DLPFC (*p* < 0.001), right DLPFC (*p* < 0.001), left PMC (*p* < 0.001), right PMC (*p* < 0.001), and left S1 (*p* < 0.001). However, no significant difference in ΔHbO values was noted at right S1. The observed differences were all statistically significant (Table III and Fig. 3).

**Table III:** Paired comparison

fNIRS	Grasping task	Handbike task	Between-group comparison
Left SM1 (HbO mmol/L)	0.050 (0.008, 0.051)	0.039 (0.008, 0.048)	*p* = 0.048
Right SM1 (HbO mmol/L)	0.046 (0.006, 0.051)	0.045 (0.008, 0.056)	*p* < 0.001
Left DLPFC (HbO mmol/L)	0.034 (0.010, 0.038)	0.012 (0.005, 0.021)	*p* < 0.001
Right DLPFC (HbO mmol/L)	0.037 (0.014, 0.042)	0.017 (0.006, 0.026)	*p* < 0.001
Left PMC (HbO mmol/L)	0.043 (0.006, 0.045)	0.037 (0.007, 0.043)	*p* < 0.001
Right PMC (HbO mmol/L)	0.037 (0.008, 0.040)	0.034 (0.007, 0.039)	*p* < 0.001
Left S1 (HbO mmol/L)	0.046 (0.008, 0.049)	0.039 (0.008, 0.048)	*p* < 0.001
Right S1 (HbO mmol/L)	0.042 (0.007, 0.049)	0.041 (0.007, 0.049)	*p* = 0.17

Descriptive statistic report median (interquartile range).

SM1: primary sensorimotor cortex; DLPFC: dorsolateral prefrontal cortex; PMC: primary motor cortex; S1: primary somatosensory cortex; HbO mmol/L: oxyhaemoglobin mmol per litre.

### Comparison of brain functional connectivity under grasping task and handbike task

**Fig. 4:**
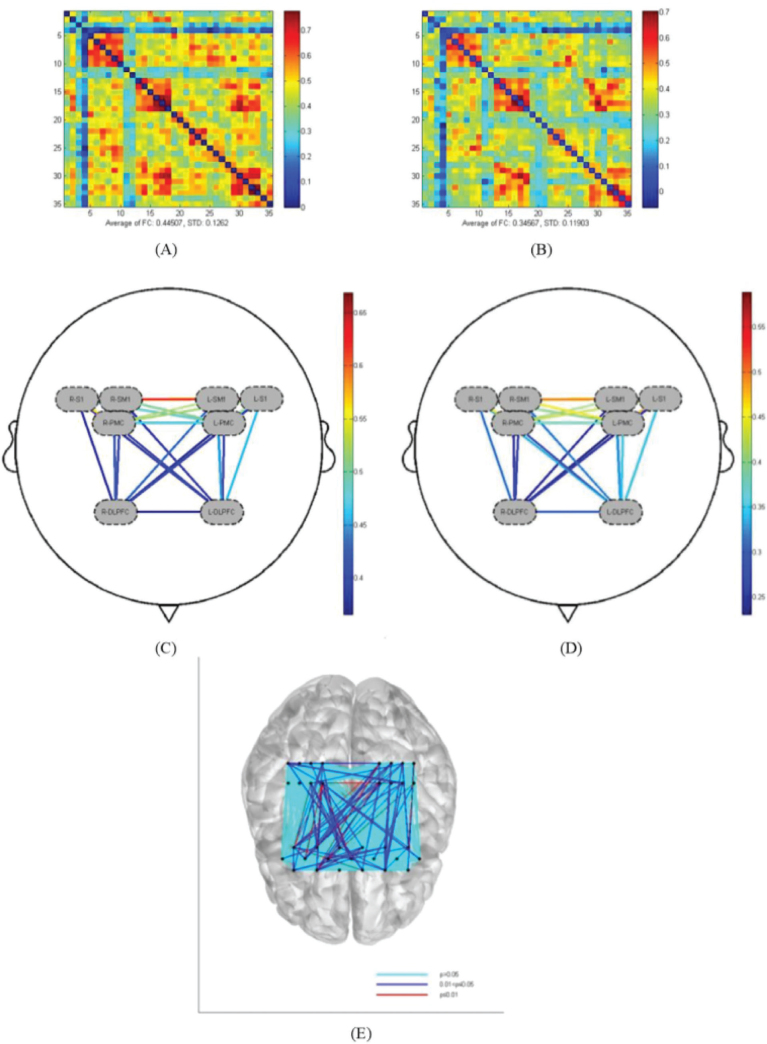
Comparison of functional connectivity between channels under different hand tasks. (A) Channel connectivity matrix for the grasping task of the affected hand. (B) Channel connectivity matrix for the handbike task. (C) ROI connectivity in the grasping task of the affected hand. (D) ROI connectivity in the handbike task. (E) Comparison of the statistical results of functional connectivity in the grasping task and the handbike task.

Fig. 4 illustrates that compared with the handbike task, the grasping task exhibited stronger functional connectivity between right DLPFC and bilateral PMC, left S1 (*p* < 0.05), and bilateral SM1 (*p* < 0.05). Additionally, stronger functional connectivity was observed within the right DLPFC (*p* < 0.05). All observed differences were statistically significant (Table IV).

**Table IV:** Statistical comparison of functional connectivity between grasping task and handbike task

fNIRS	Grasping task	Handbike task	Between-group comparison	T value
Right DLPFC-left SM1	0.422 ± 0.238	0.257 ± 0.275	*p* = 0.005	3.062
Right DLPFC-right SM1	0.406 ± 0.265	0.280 ± 0.291	*p* = 0.040	2.150
Right DLPFC-left PMC	0.372 ± 0.249	0.233 ± 0.256	*p* = 0.001	2.862
Right DLPFC-right PMC	0.419 ± 0.241	0.294 ± 0.249	*p* = 0.007	2.924
Right DLPFC-left S1	0.399 ± 0.251	0.277 ± 0.274	*p* = 0.027	2.329
Right DLPFC-right DLPFC	0.386 ± 0.252	0.262 ± 0.235	*p* = 0.019	2.485

Descriptive statistic report median (interquartile range).

SM1: primary sensorimotor cortex; DLPFC: dorsolateral prefrontal cortex; PMC: primary motor cortex; S1: primary somatosensory cortex; HbO mmol/L: oxyhaemoglobin mmol per litre.

## DISCUSSION

In this study, cortical activation and functional connectivity measured by fNIRS were employed to analyse the neural effects of stroke patients with hand dysfunction during different hand tasks. Results indicate variations in the activation of the central nervous system among these patients across different tasks. Both the grasping task and handbike task significantly activated the cognitive, motor, and sensory cortex of the brain. Compared with the hand-crank cycling task, the grasping task elicited stronger and more widespread cortical activation, which aligns with the theory that motor tasks with higher sequence complexity and greater fine motor demands activate more extensive and asymmetrical cortical networks. The grasping task requires precise finger coordination and complex action sequencing, thereby imposing greater demands on motor planning and execution systems than the bilateral, coordinated, and assisted hand-cranking movements. These findings suggest that task selection may play an important role in guiding and optimizing therapeutic rehabilitation programmes. Grasping exercises and handbike exercises are commonly used rehabilitation methods for hand dysfunction following stroke. They serve as task paradigms to evaluate treatment effects in experimental studies. Grasping movement is currently the predominant paradigm utilized by fNIRS to assess hand motor function in stroke patients. Several studies have evaluated the haemodynamic response of the motor cortex through grasping movements, finger movements, or keyboard-tapping paradigms in stroke patients (22). Previous research has indicated that the whole-hand fist-clenching task elicits greater cortical activation than classical motor tasks such as finger tapping (23). Additionally, some studies have suggested that there is no significant difference in motor cortex activation between these 2 motor task paradigms, indicating their interchangeability in clinical fNIRS research (24). The handbike task is a comprehensive upper limb movement paradigm assisted by a robotic arm, requiring patients to exhibit stable grip with the affected hand and perform upper limb flexion and extension. In this study, fNIRS was employed for dynamic monitoring in patients with hand dysfunction following stroke during resting state, active hand grasping task, and 2-handed hand-rolling bicycle task, aiming to elucidate differences in brain activation and functional connectivity patterns across different task states.

Previous studies have indicated that exercise training can enhance both motor and cognitive function, with reciprocal influences between the 2 domains, although the neural mechanisms following stroke remain unclear (25). The active grasping movement of the affected hand engages not only motor-related functions such as action execution but also cognition-related functions such as motor planning and attention resource allocation (26). Yeo et al. (27) demonstrated that activation during shoulder movements involves contralateral premotor areas and supplementary motor cortex, which are more medial and extensive compared with hand movements. Bonnal et al. (28) found that hand grasping movement in healthy subjects is associated with contralateral SM1 activation. Additionally, an fMRI study by Hannanu et al. (29) revealed activity in the sensorimotor cortex and parietal lobe during the grasping task. Our results indicate that affected hand-grasping movement in stroke patients is associated with bilateral SM1 activation, suggesting an active role of both hemispheres in restoring interhemispheric excitation–inhibition balance and in the planning and execution of voluntary movements. Furthermore, our brain network functional connectivity data demonstrate that motor training strengthens connections between cognitive networks and motor networks, as well as within cognitive networks. Specifically, we observed greater connectivity between the right DLPFC and bilateral PMC, bilateral SM1, and left S1 in the grasping group. These areas are known to be involved in conscious motor control. Therefore, this study offers a cortical haemodynamic rationale for the enhancement of motor and cognitive function through exercise training. The recovery of grasping ability post-stroke precedes that of single-finger movement, suggesting its suitability for early stroke rehabilitation training. Spasticity, a common post-stroke complication, significantly impairs motor function due to persistent muscle hypertonia. Consequently, patients must overcome flexor tension to achieve finger extension during the affected hand grasping task, necessitating greater motor planning and cognitive and motor resources. In comparison, the handbike task requires less muscle tone and fine motor control, leading to relatively lower central nervous system resource mobilization. However, this may also relate to diminished patient initiative and engagement with exercise training when aided by equipment. The handbike task, being assisted by equipment, involves a relatively straightforward movement trajectory for the affected hand, requiring no additional movement planning and lower utilization of cognitive resources. Moreover, it can be conceptualized as a progressive automation process, allowing task performance without sustained attentional control, possibly contributing to the relatively lower activation observed during the handbike task.

Functional connectivity (FC) quantifies the temporal correlation of neurophysiological events in spatially distinct brain regions, revealing the functional interactions of specific brain regions and local networks (30). Previous studies have demonstrated that interhemispheric FC decreases in behavioural areas such as motor function, attention, memory, and vision after stroke, suggesting impaired interhemispheric communication as a key feature of stroke (31). The results of this study confirm the regulatory effect of hand grasping and handbike training on interregional information exchange difficulty in stroke patients, as well as the recovery of motor planning and executive function, which promotes the repair of neurons on the affected side and facilitates functional network remodelling of brain regions associated with motor and cognition.

This research is an exploratory study. Before formal acquisition, a pre-measurement phase was conducted to ensure that all channels achieved optimal signal quality. During pre-processing, wavelet-based methods were applied to remove motion-related and environmental artefacts. Furthermore, band-pass filtering (0.01–0.1 Hz) was used to eliminate physiological noise, including cardiac, respiratory, and Mayer wave signals (32, 33). These procedures collectively enhanced the reliability of the fNIRS measurements to a certain extent.

However, this study has several limitations. First, the sample size was small. Second, there was no stratified analysis based on the location and severity of injury in stroke patients with hand dysfunction. Third, whether wearing a device-assisted movement device with similar single-finger distance, height, and degree of finger flexion reduces cortical activation in the affected hand grip task compared with full voluntary movement warrants further investigation. Fourth, the patients included in this study were enrolled on average 8.1 weeks after stroke. Although this time window covers an important phase of rehabilitation, it may miss the period when spontaneous neuroplasticity is most active immediately after stroke. Future studies including patients in the hyperacute and acute phases (< 2 weeks) will help clarify the patterns during this stage. Fifth, a limitation of this study is that the enrolled participants had a wide range of post-stroke durations (2–24 weeks), and the study did not conduct subgroup analyses based on the length of post-stroke duration. In future studies, we plan to recruit a larger cohort to enable subgroup analyses according to disease duration. Sixth, this study did not explicitly analyse the potential impact of the dominant hand on cortical activation patterns. Considering the significant influence of the dominant hand on cognitive function, this study did not account for the differences in motor execution caused by the dominant hand. Future research should control for this factor and conduct more in-depth studies. Moreover, the paradigm of this study focused on comparing the 2 tasks performed by the affected limb. Seventh, we did not include a control condition in which the same grasping task was performed by the unaffected hand. Including such a condition in future studies would be valuable for directly assessing interhemispheric inhibition, activation spillover from the unaffected hemisphere, and the genuine lateralization process during recovery. Eighth, strong mobility and good time resolution are 2o advantages of fNIRS over fMRI. Nevertheless, fNIRS’s penetration depth is restricted to the cerebral cortex and it is unable to identify subcortical regions. Nonetheless, this study involving multiple regions of the cerebral cortex provides novel insights for the rehabilitation of hand dysfunction in stroke patients.

Future large-sample, multicentre longitudinal intervention studies could utilize the cortical activation patterns identified across different task paradigms (affected-hand grasping, hand-crank cycling) as biomarkers. These studies should evaluate their predictive value for rehabilitation outcomes, identifying which patients with specific cortical activation patterns are better suited for rehabilitation programmes emphasizing grasping training vs hand-crank cycling. This approach would enable personalized rehabilitation strategies.

In conclusion, both hand grasping and handbike exercises effectively activated the cognitive, sensory, and motor cortex in stroke patients. However, hand grasping tasks elicited stronger activation and functional connectivity in brain regions associated with hand movement compared with handbike tasks. These findings offer valuable insights for selecting upper limb rehabilitation programmes for stroke patients with hand dysfunction, guiding the development of rehabilitation protocols, and informing future fNIRS studies.

## Supplementary Material




