# Supplementary material for: RECRUITMENT OF THE CENTRAL NERVOUS SYSTEM IN DIFFERENT HAND TASKS IN PATIENTS WITH HAND DYSFUNCTION AFTER STROKE BASED ON FUNCTIONAL NEAR-INFRARED SPECTROSCOPY: AN EXPLORATORY STUDY
Source: J Rehabil Med. 2026 Mar 9;58:44712. doi: 10.2340/jrm.v58.44712 (PMC12980317; doi:10.2340/jrm.v58.44712)
Supplement: Supplementary file 1 [file JRM-58-44712-s1.pdf]

Supplementary material has been published as submitted. It has not been copyedited, or typeset by Journal of Rehabilitation Medicine

Table S1. Four ROIs related to hand movement

ID	Region	Abbreviation	Side	Channel
1	Primary sensorimotor cortex	SM1	L	14 32 33
			R	16 17 30
2	Dorsolateral prefrontal cortex	DLPFC	L	9 11 12 24 25 26 27
			R	3 4 5 19 20 21 23
3	Primary motor cortex	PMC	L	28 29 34 35
			R	1 15 18 31
4	Primary somatosensory cortex	S1	L	13 14 32
			R	2 16 17

Table S2. Comparison of Brain Area Activation Between Resting State and Grasping Task.

ROIs	Task	M(P25，P75)	Wilcoxon's two samples rank sum test Z value	P value
left SM1	rest state	0.016(0.011，0.019)	18.892	P<0.001
	grasping task	0.050(0.008，0.051)		
right SM1	rest state	0.012(0.007，0.015)	19.208	P<0.001
	grasping task	0.046(0.006，0.051)		
left DLPFC	rest state	0.006(0.002，0.009)	21.236	P<0.001
	grasping task	0.034(0.010，0.038)		
right DLPFC	rest state	0.021(0.015，0.024)	17.381	P<0.001
	grasping task	0.037(0.014，0.042)		
left PMC	rest state	-0.001(-0.005，0.002)	22.238	P<0.001
	grasping task	0.043(0.006，0.045)		
right PMC	rest state	0.007(0.004，0.011)	18.493	P<0.001
	grasping task	0.037(0.008，0.040)		
left S1	rest state	0.016(0.010，0.019)	19.145	P<0.001
	grasping task	0.046(0.008，0.049)		
right S1	rest state	0.010(0.005，0.013)	19.684	P<0.001
	grasping task	0.042(0.007，0.049)		

Table S3. Comparison of Brain Area Activation Between Resting State and handbike Task.

ROIs	Task	M(P25，P75)	Wilcoxon's two samples rank sum test	
			Z value	P value
left SM1	rest state	0.016(0.011，0.019)	17.331	P<0.001
	handbike task	0.039(0.008，0.048)		
right SM1	rest state	0.012(0.007，0.015)	19.938	P<0.001
	handbike task	0.045(0.008，0.056)		
left DLPFC	rest state	0.006(0.002，0.009)	9.974	P<0.001
	handbike task	0.012(0.005，0.021)		
right DLPFC	rest state	0.021(0.015，0.024)	7.284	P<0.001
	handbike task	0.017(0.006，0.026)		
left PMC	rest state	-0.001(-0.005，0.002)	21.576	P<0.001
	handbike task	0.037(0.007，0.043)		
right PMC	rest state	0.007(0.004，0.011)	18.040	P<0.001
	handbike task	0.034(0.007，0.039)		
left S1	rest state	0.016(0.010，0.019)	17.709	P<0.001
	handbike task	0.039(0.008，0.048)		
right S1	rest state	0.010(0.005，0.013)	19.617	P<0.001
	handbike task	0.041(0.007，0.049)		
