# Supplementary material for: RECRUITMENT OF THE CENTRAL NERVOUS SYSTEM IN DIFFERENT HAND TASKS IN PATIENTS WITH HAND DYSFUNCTION AFTER STROKE BASED ON FUNCTIONAL NEAR-INFRARED SPECTROSCOPY: AN EXPLORATORY STUDY
Source: J Rehabil Med. 2026 Mar 9;58:44712. doi: 10.2340/jrm.v58.44712 (PMC12980317; doi:10.2340/jrm.v58.44712)
Supplement: Supplementary file 2 [file JRM-58-44712-s2.pdf]

Supplementary material has been published as submitted. It has not been copyedited, or typeset by Journal of Rehabilitation Medicine

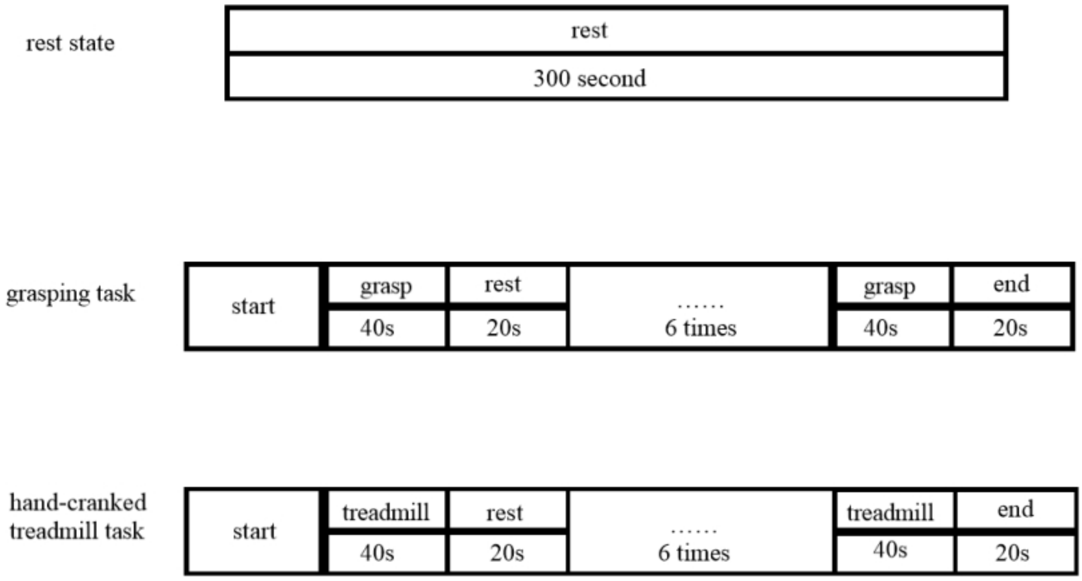

Figure S1. fNIRS measurement paradigm.

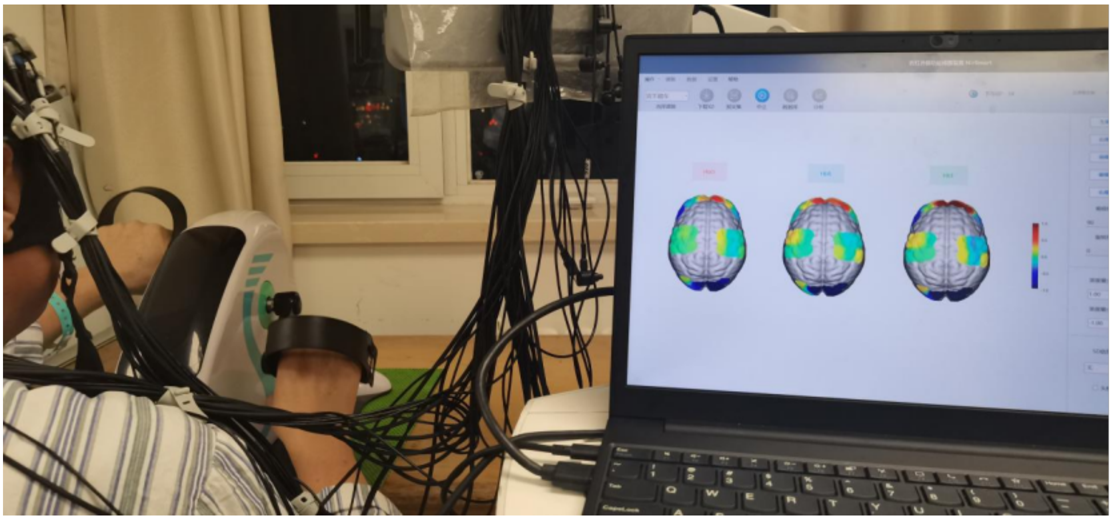

Figure S2. handbike fNIRS measurement.
